# Use of a specialized peptide-based enteral formula containing medium-chain triglycerides for enteral tube feeding in children with cerebral palsy and previous tube feeding intolerance on standard enteral formula: a prospective observational TolerUP study

**DOI:** 10.3389/fped.2025.1448507

**Published:** 2025-02-12

**Authors:** Aydan Kansu, Gunsel Kutluk, Gonul Caltepe, Cigdem Arikan, Nafiye Urganci, Gokhan Tumgor, Aysel Yuce, Ceyda Tuna Kirsaclioglu, Arzu Meltem Demir, Fatma Demirbas, Merve Usta, Sibel Yavuz, Duygu Demirtas Guner, Ersin Gumus, Buket Dalgic, Yasar Dogan, Nelgin Gerenli, Halil Kocamaz, Fulya Gulerman, Elif Sag, Aysugul Alptekin Sarioglu, Neslihan Eksi Bozbulut, Demet Teker Duztas, Hatice Altug Demirol, Coskun Celtik, Olcay Gungor, Kaan Demiroren, Aysen Uncuoglu Aydogan, Ozlem Bekem, Zeynep Arslan, Murat Cakir, Arzu Ekici, Nihal Uyar Aksu, Cigdem Ecevit, Simge Erdogan

**Affiliations:** ^1^ Department of Pediatric Gastroenterology, Ankara University School of Medicine, Ankara, Türkiye; ^2^ Department of Pediatric Gastroenterology, Kanuni Sultan Suleyman Training and Research Hospital, Istanbul, Türkiye; ^3^ Department of Pediatric Gastroenterology, Ondokuz Mayis University Faculty of Medicine, Samsun, Türkiye; ^4^ Department of Pediatrics, Division of Pediatric Gastroenterology, Hepatology and Nutrition, Koc University Faculty of Medicine, Istanbul, Türkiye; ^5^ Department of Pediatric Gastroenterology, Sisli Hamidiye Etfal Training and Research Hospital, Istanbul, Türkiye; ^6^ Department of Pediatrics, Division of Pediatric Gastroenterology, Hepatology and Nutrition, Cukurova University Faculty of Medicine, Adana, Türkiye; ^7^ Department of Pediatrics, Division of Pediatric Gastroenterology, Hepatology and Nutrition, Hacettepe University Faculty of Medicine, Ankara, Türkiye; ^8^ Department of Pediatric Gastroenterology, Ankara City Hospital, Ankara, Türkiye; ^9^ Department of Pediatric Gastroenterology, Gazi University Faculty of Medicine, Ankara, Türkiye; ^10^ Department of Pediatric Gastroenterology, Firat University Faculty of Medicine, Elazig, Türkiye; ^11^ Department of Pediatric Gastroenterology, Istanbul Umraniye Training and Research Hospital, Istanbul, Türkiye; ^12^ Department of Pediatrics, Division of Pediatric Gastroenterology, Pamukkale University Faculty of Medicine, Denizli, Türkiye; ^13^ Department of Pediatric Gastroenterology, Kirikkale University Faculty of Medicine, Kirikkale, Türkiye; ^14^ Department of Pediatric Gastroenterology Hepatology and Nutrition, Karadeniz Technical University Faculty of Medicine, Trabzon, Türkiye; ^15^ Abbott Laboratories, Istanbul, Türkiye; ^16^ Department of Pediatric Gastroenterology, Bursa Yuksek Ihtisas Training and Research Hospital, Bursa, Türkiye; ^17^ Department of Pediatrics, Division of Pediatric Gastroenterology, Hepatology and Nutrition, Kocaeli University Faculty of Medicine, Kocaeli, Türkiye; ^18^ Department of Pediatrics, Division of Pediatric Gastroenterology, Hepatology and Nutrition, University of Health Sciences Dr. Behcet Uz Children's Hospital, Izmir, Türkiye; ^19^ Department of Pediatric Neurology, Bursa Yuksek Ihtisas Training and Research Hospital, Bursa, Türkiye

**Keywords:** cerebral palsy, peptide-based formula, gastrointestinal intolerance, anthropometrics, parental satisfaction

## Abstract

**Objective:**

Use of peptide-based formulas supplemented with medium chain triglycerides (MCTs) is considered a beneficial strategy to decrease the tube-feeding associated gastrointestinal tolerance. In children with cerebral palsy (CP), overall effects of enteral tube feeding as well as the utility of peptide-based specialized enteral formulas in those with gastrointestinal intolerance have not been extensively studied. This study aimed to evaluate the utility of enteral tube feeding via specialized peptide-based formula containing MCTs in children with CP in terms of gastrointestinal intolerance, anthropometrics, defecation characteristics and parental satisfaction with enteral formula.

**Methods:**

Children with CP who received enteral tube feeding via specialized peptide-based formula containing MCTs were included in this prospective observational study. Anthropometrics (*z* scores for weight for age [WFA], weight for height [WFH], triceps skinfold thickness [TSFT] and mid-upper arm circumference [MUAC]), gastrointestinal intolerance symptoms, defecation frequency and stool patterns and formula satisfaction were recorded at baseline and during 6-month follow up.

**Results:**

A total of 96 children with CP (mean ± SD age: 5.6 ± 3.2 years, 56.3% were boys) were included. Significant improvements were noted in MUAC, TSFT and WFH *z* scores at the 6th month visit. The rate of “severe symptoms” and the likelihood of Type-1/Type-2 (constipation) stool pattern were significantly decreased. Majority of parents were satisfied with the study formula.

**Conclusion:**

Our findings revealed favorable efficacy and safety of using a specialized peptide-based formula containing MCT in provision of enteral tube feeding among children with CP in terms of improved anthropometrics, amelioration of gastrointestinal intolerance symptoms and normalization of bowel movements along with a high parental satisfaction.

## Introduction

Functional feeding disorders and gastrointestinal dysfunction are highly prevalent conditions in children with cerebral palsy (CP), which compromise the adequate nutrient intake in these children by causing vomiting, dysphagia, impaired swallowing, gastroesophageal reflux, aspiration and constipation, in addition to limited self-feeding ability (1–5). Accordingly, many children with CP are considered at high risk of poor nutritional status, particularly those with severe gross motor impairment and oropharyngeal dysfunction (2, 4, 6, 7).

In children with CP, malnutrition leads to growth failure, decreased cerebral function and reduced potential for development, impaired immune, respiratory and gastro-intestinal functions, delayed wound healing, increased morbidity and reduced quality of life (8–10).

Owing to increased life-expectancy in the presence of disability, the prevalence and consequences of feeding difficulties are on the rise in children with CP, making nutritional status an indispensable part of the medical care (9–11). Hence, comprehensive evaluation and treatment of feeding disorders via adequate nutritional support based on better tolerated enteral formulas is considered essential in improving the nutritional status, anthropometrics, energy store and quality of life, as well as in decreasing irritability, spasticity and hospitalization rates in neurologically impaired children including those with CP (12–14).

Enteral tube feeding, via nasogastric or gastrostomy tube feeding depending on the anticipated duration of enteral nutrition, is frequently indicated in children with CP with significant oropharyngeal incoordination who are unable to meet their nutritional requirements orally (11, 12). However, feeding intolerance and related complications comprise a major drawback to enteral tube feeding in children with severe impairment of the central nervous system (15). The formula composition, proteins in particular, is considered critical in provision of best nutritional support in children with CP, given the persisting feeding difficulties in gastrostomy-fed children in addition to adverse effects of highly prevalent gastrointestinal symptoms (possibly related to delayed gastric emptying and gastric dysmotility) on the already impaired nutritional status (1, 16–18).

The higher efficacy and better tolerability of peptide-based formulas, as compared with formulas composed entirely of free amino acids or intact protein, have been reported in malnourished patients, particularly in those with gastrointestinal dysfunction and thus inability to tolerate nutritional supplements containing whole protein or long chain triglycerides (19–21). Patients with impaired gastrointestinal function are considered to require a formula containing hydrolyzed protein and medium chain triglycerides (MCTs) to minimize the need for hydrolysis of protein by the intestinal brush border peptidases for an easier absorption (19, 20, 22). Hence, although being addressed by only a few studies in critically ill patients, use of formulas that are peptide-based and supplemented with MCT and fiber is considered amongst the potential nutritional interventions to decrease the tube-feeding associated gastrointestinal tolerance (21). The overall effects of enteral tube feeding as well as the utility of peptide-based specialized enteral formulas in those with gastrointestinal intolerance have not been extensively studied among children with CP (6, 23–25).

This prospective observational study was designed to evaluate the utility of a specialized peptide-based formula containing MCT in children with CP who had previous tube feeding intolerance on standard enteral formula, in terms of effects on gastrointestinal intolerance, anthropometrics, defecation frequency and stool patterns and parental satisfaction with enteral formula.

## Materials and methods

### Study population

Children with CP who received enteral tube feeding with a specialized peptide-based enteral formula containing partially hydrolyzed protein plus MCTs, due to previous tube feeding intolerance on standard enteral formula, were included in this prospective observational multi-center 6-month follow up study conducted at 17 centers across Turkey. Hospitalized or outpatient children with CP (1–12 years of age) who developed gastrointestinal intolerance on enteral tube feeding with standard enteral formula and therefore were planned to receive a specialized peptide-based enteral formula that contain partially hydrolyzed protein plus MCTs were included in the study. Presence of another chronic disease (diabetes, liver disease, kidney disease, endocrine disease, cancer), intestinal obstruction, need for dialysis treatment, artificial respiratory device, parenteral nutrition or normal nutrition in addition to enteral tube feeding were the exclusion criteria of the study. Although 126 children with CP were initially enrolled in the study, 96 children comprised the final study population, since 30 children were excluded due to protocol violation (*n* = 13), adverse events (*n* = 7), death (*n* = 6), dissatisfaction with the nutritional product (*n* = 2), withdrawal of informed consent form (*n* = 1) and other reasons (*n* = 1).

Written informed consent was obtained from parent/legal guardian of each subject. The study was conducted in accordance with the ethical principles stated in the “Declaration of Helsinki” and approved by the Ankara University Faculty of Medicine Clinical Research Ethics Committee (Date of Approval: 24/07/2017; Protocol No: 12-736-17) and the Republic of Turkey Ministry of Health Turkish Medicines and Medical Devices Agency (Date of approval: 16/08/2017; Protocol No: 93189304-514.05.01-E.168888).

### Assessments

Data on demographic characteristics (age, gender), clinical and functional subtypes of CP and gastrointestinal intolerance symptoms were recorded at baseline. Data on clinical nutrition characteristics (type, route, mode of delivery, daily volume), total energy expenditure (TEE), anthropometrics (body mass index [BMI], weight for age [WFA], weight for height [WFH], triceps skinfold thickness [TSFT] and mid-upper arm circumference [MUAC]) expressed as *z* scores, gastrointestinal intolerance symptoms (severity and frequency), and defecation frequency and stool patterns were recorded at baseline and at three consecutive visits (1st, 3rd and 6th months) during a 6-month follow up. Parent's assessment on feeding and stool patterns and formula satisfaction were also evaluated via responses to the Feeding and Stool Patterns Questionnaire (at baseline and 1st, 3rd and 6th months) and Formula Satisfaction Questionnaire (at 1st, 3rd and 6th months), respectively, which were applied through face-to-face interview method.

### Anthropometry

Body weight was measured using a digital baby weight scale (10 g precision) in children aged ≤2 years, while with adult electronic scale (100 g precision) in children aged >2 years. Length measurement was performed using a 1 m length measuring tape (0.1 cm precision) in children aged ≤2 years, with a wall mounted stature meter (0.2 cm precision) in children aged >2 years, and with the use of tibial length (measured via a flexible tape measure from the superior border of the medial tibia condyle to the inferior border of the medial malleolus, with both the knee and the ankle at 90 degrees) for the stature estimations when necessary (26). MUAC was measured from the left upper arm flexed slightly at the elbow, at half distance between the acromion and the olecranon using a plastic measuring tape. TSFT was measured from the left arm, and at half distance between the acromion and the olecranon, using a skin fold caliper. Anthropometric data were expressed as *z* scores for BMI, TSFT and MUAC along with estimation of mean z-scores and percentiles for WFA and WFH (27). Growth was assessed based on the maintenance of anthropometric z-scores (using WHO reference data) during the study (28).

### Gastrointestinal intolerance and defecation

Gastrointestinal intolerance symptoms were evaluated in terms of frequency and severity. Symptom frequency scores ranged from 1 to 6 (1: none, 2: once a month, 3: once a week, 4: 2–6 times in a week, 5: 1–2 times per day and 6: ≥3 times per day). Symptom severity scores were grouped as score 1–2 (none-mild), score 3 (moderate) and score 4–5 (severe).

Defecation frequency was evaluated in “at least 2–3 times per day”, “once a day/once in 2–3 days”, “once a week or less” groups. The stool patterns were evaluated using Bristol Scale as categorized into type 1–2 (indicate constipation), type 3–4 (ideal stools as they are easier to pass), and type 5–7 (indicate diarrhea and urgency) (29).

### TEE

TEE (kcal/day) was estimated using the height-based method for energy requirements of children with CP, including 15 kcal/cm in children without motor dysfunction, 14 kcal/cm for ambulatory children having motor dysfunction and 11 kcal/cm for non-ambulatory children (30).

### Statistical analysis

Statistical analysis was performed using IBM SPSS Statistics for Windows, Version 20.0 (IBM Corp., Armonk, NY). The numerical data were analyzed using Friedman test (with pairwise comparisons via Wilcoxon test) where the repeated measurement statistics did not comply with the parametric test assumptions, while repeated measurement statistics were used otherwise. Change over time was evaluated by Wilcoxon test for non-normally distributed variables. Data were expressed as mean ± standard deviation (SD), median (minimum–maximum) and percent (%) where appropriate. *p* < 0.05 was considered statistically significant.

## Results

### Baseline characteristics

A total of 96 children with CP were included in the study. The mean age of children with CP was 5.6 years (SD 3.2, range 1.0–12.0 years), and 56.3% of children were boys. Children with spastic CP (78.0%) and severe (30.4%) or total (20.7%) functional limitation comprised the majority of study population. The most common gastrointestinal intolerance symptoms recorded at baseline were gastrointestinal discomfort (65.6%), constipation (62.5%), retching (58.3%), nausea (52.8%), gas (49.5%), stomachache (42.4%) reflux/regurgitation (40.0%) and abdominal distention (37.2%) (Table 1).

**Table 1:** Baseline characteristics in children with CP.

Patient demographics		
Age (year)	Mean ± SD	5.6 ± 3.2
	Median (min–max)	5.0 (1.0–12.0)
Gender, *n* (%)		
Girl		42 (43.8)
Boy		54 (56.3)
CP classification, *n* (%)		
Clinical classification		
Spastic		71 (78.0)
Hemiplegia		5 (7.0)
Diplegia		3 (4.2)
Quadriplegia		63 (88.7)
Extrapyramidal		4 (4.4)
Rigidity		3 (75.0)
Ataxia		1 (25.0)
Mixed		16 (17.6)
Primary spastic		14 (87.5)
Primary extrapyramidal		2 (12.5)
Functional classification		
No activity limitation		12 (13.0)
Mild activity limitation		33 (35.9)
Severe activity limitation		28 (30.4)
No functional activity		19 (20.7)
Gastrointestinal intolerance symptoms at enrolment		
Discomfort		63 (65.6)
Constipation		60 (62.5)
Retching		56 (58.3)
Nausea		47 (52.8)
Gas		47 (49.5)
Stomachache		28 (42.4)
Reflux/Regurgitation		38 (40.0)
Abdominal distention		35 (37.2)
Vomiting		32 (33.3)
Residue		10 (15.2)
Diarrhea		13 (13.5)

### Peptide-based enteral formula characteristics

Majority of children received Pediasure peptide® 1.0 Cal [Abbott Nutrition; hydrolyzed whey-dominant protein; 7 g (14% E) per 100 ml] at baseline (90.6%, via PEG in 88.5% and via NGT in 11.5%) as well as in the 3rd month (89.6%) and 6th month (92.7%) visits. Peptisorb® [100% whey protein; 2.8 g (11% E) per 100 ml] users comprised the 9.4% (baseline and 3rd month) and 7.3% (6th month) of study population (Table 2). Mode of delivery included bolus injection in 59.6% of children, while pump-assisted administration and gravity-controlled administration were applied in 34.0% and 6.5% of children, respectively. Median daily total volume was 800 ml (61.5 ml/kg) at baseline and 1,000 ml (63.5 ml/kg) at the 6th month visit (Table 2).

**Table 2:** Nutritional characteristics in children with CP.

		Nutritional characteristics at baseline		
		Overall	PEG	NGT
Peptide-based enteral formula, *n* (%)	Pediasure® peptide	87 (90.6)	77 (88.5)	10 (11.5)
	Peptisorb®	9 (9.4)	9 (100.0)	0 (0.0)
Mode of delivery, *n* (%)				
Pump-assisted		32 (34.0)	29 (90.6)	3 (9.4)
Gravity-controlled		6 (6.4)	3 (50.0)	3 (50.0)
Bolus injection		56 (59.6)	53 (94.6)	3 (5.4)
Duration of use (month), median (min–max)		11.0 (0.10–96.0)	12.0 (0.5–96.0)^a^	9.0 (0.10–50.0)^b^
		Nutritional characteristics at study visits		
		Baseline	3rd month	6th month
Peptide-based enteral formula, *n* (%)	Pediasure® peptide	87 (90.6)	86 (89.6)	89 (92.7)
	Peptisorb®	9 (9.4)	9 (9.4)	7 (7.3)
	Bebelac pepti junior®	—	1 (1)	—
Daily total volume, median (min–max)	ml/day	800 (240–1,800)	918 (250–1,800)	1,000 (275–2,000)
	ml/kg	61.5 (21.1–153.8)	65.5 (21.8–148.1)	63.5 (21.7–166.7)
TEE (kcal/day), *n* (%)				
15 kcal/cm (no motor dysfunction)		2 (2.1)	3 (3.2)	3 (3.2)
14 kcal/cm (mobile with motor dysfunction)		24 (25.5)	18 (19.1)	14 (14.7)
11 kcal/cm (immobile)		68 (72.3)	73 (77.7)	78 (82.1)
Daily total volume by TEE, median (min–max)				
ml/day	15 kcal/cm (no motor dysfunction)	1,270 (1,000–1,540)	1,400 (1,200–1,800)	1,200 (1,000–1,400)
	14 kcal/cm (mobile with motor dysfunction)	960 (480–1,600)	960 (420–1,600)	1,140 (800–2,000)
	11 kcal/cm (immobile)	800 (240–1,800)	900 (250–1,800)	1,000 (275–1,540)
ml/kg	15 kcal/cm (no motor dysfunction)	78.1 (27.8–128.3)	92.3 (47.4–100)	83.3 (33.4–98.6)
	14 kcal/cm (mobile with motor dysfunction)	69.1 (27.6–153.8)	72.9 (36.4–148.1)	73.3 (22.7–139.3)
	11 kcal/cm (immobile)	60.3 (21.1–133.3)	64.4 (21.8–142.9)	61.1 (21.7–166.7)

NGT, nasogastric tube; PEG, percutaneous endoscopic gastrostomy; TEE, total energy expenditure.

The duration of use is not specified for ^a^16 patients and ^b^1 patient.

Immobile children with 11 kcal/cm height/day energy need comprised the majority of study population in each follow up visit (72.3%, 77.7% and 82.1%, respectively). In these children, the median daily total volume was 800 ml (60.3 ml/kg) at baseline and 1,000 ml (61.1 ml/kg) at 6th month visit (Table 2).

### Anthropometrics at baseline and follow up visits

**Figure 1:**
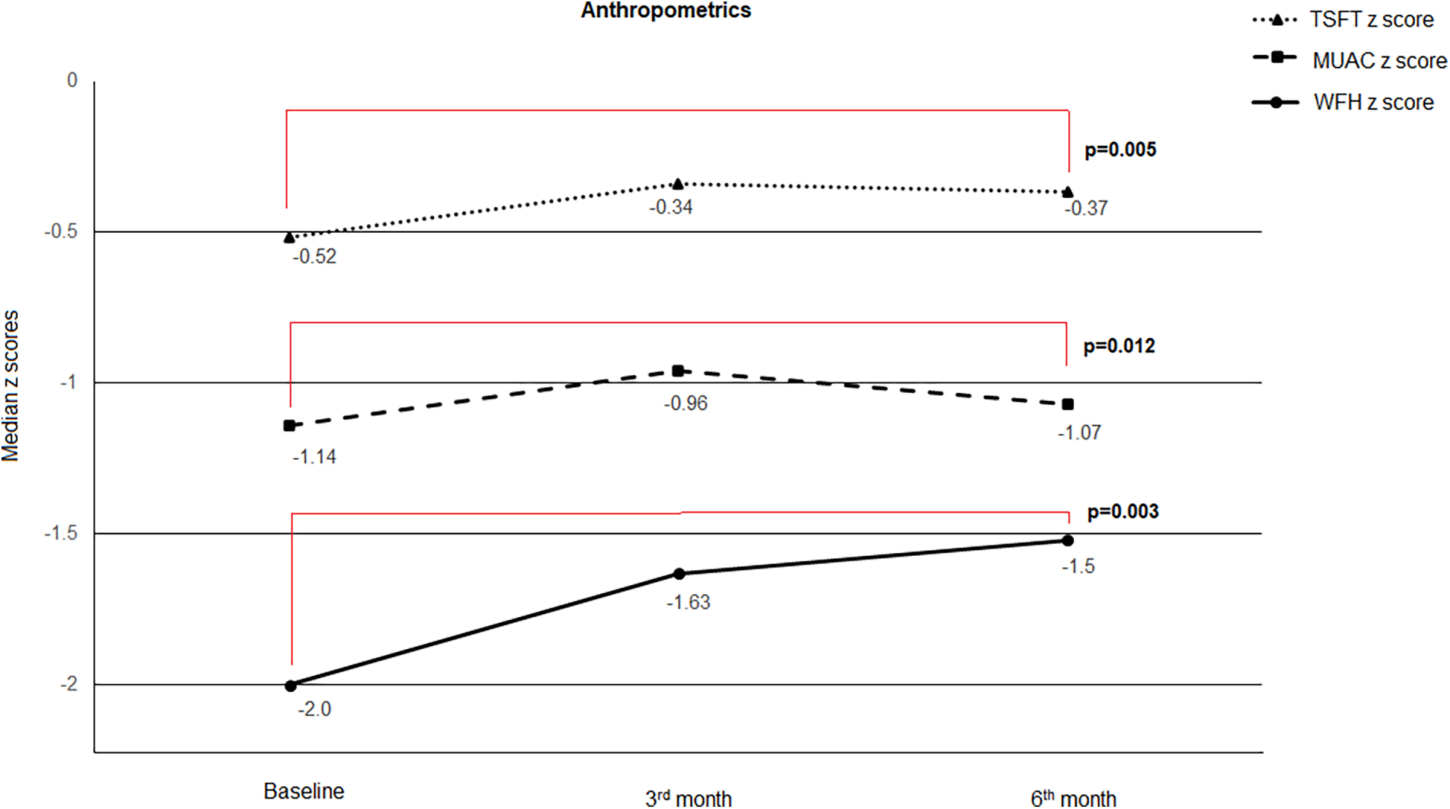
Change in anthropometric *z* scores during study visits. When compared to baseline scores, significant improvement was noted in median (min/max) MUAC *z* scores [−1.14(−2.68/−0.19) vs. −1.07(−2.07/0.32), *p* = 0.012], TSFT *z* scores [−0.52 (−1.29/0.40) vs. −0.37(−1.05/0.92), *p* = 0.005] and WFH *z* scores [−2.0 (−3.40/−0.36) vs. −1.52 (−3.11/−0.35), *p* = 0.003] at 6th month visit.

When compared to baseline scores, significant improvement was noted in median (min/max) MUAC *z* scores [−1.14 (−2.68/−0.19) vs. −1.07 (−2.07/0.32), *p* = 0.012], TSFT *z* scores [−0.52 (−1.29/0.40) vs. −0.37 (−1.05/0.92), *p* = 0.005] and WFH *z* scores [−2.0 (−3.40/−0.36) vs. −1.52 (−3.11/−0.35), *p* = 0.003] at 6th month visit. The change from baseline MUAC *z* score was also significantly higher at 3rd month visit compared to 6th month visit [0.99 (−10.5/3.68) vs. 0.27 (−8.41/4.38), *p* = 0.012] (Table 3, Figure 1).

**Table 3:** Change in anthropometric *z* scores during study visits in children with CP who received a specialized peptide-based enteral formula.

Median (min–max)		Baseline	3rd month	6th month	*p* value
WFA *z* score (*n* = 78)	Visit score	−2.06 (−3.41/−0.68)	−2.23 (−3.50/−0.54)	−2.17 (−3.44/−0.60)	0.239^1^
	Change from baseline		0.09 (−2.9/4.04)	0.16 (−2.63/5.10)	0.365^2^
WFH *z* score (*n* = 90)	Visit score	−2.0 (−3.40/−0.36)	−1.63 (−3.14/−0.50)	−1.52 (−3.11/−0.35) ***	0.360^1^^,^^3^
	Change from baseline		0.06 (−3.53/3.98)	0.16 (−2.57/6.09)	0.340^2^
BMI *z* score (*n* = 89)	Visit score	−1.3 (−2.9/0.0)	−1.42 (−3.14/−0.06)	−1.14 (−3.50/0.14)	0.915^1^
	Change from baseline		0.09 (−4.92/5.81)	0.00 (−5.40/5.84)	0.936^2^
MUAC *z* score (*n* = 76)	Visit score	−1.14(−2.68/−0.19)	−0.96 (−2.11/0.27)	−1.07 (−2.07/0.32)*	**0** **.** **006** ^1^
	Change from baseline		0.99 (−10.5/3.68)	0.27 (−8.41/4.38)	**0.012** ^2^
TSFT *z* score (*n* = 82)	Visit score	−0.52 (−1.29/0.40)	−0.34 (−1.29/0.59)	−0.37 (−1.05/0.92)**	**0** **.** **028** ^1^
	Change from baseline		0.005 (−4.40/5.17)	0.21 (−4.33/5.25)	0.094^2^

WFA, weight for age; WFH, weight for height; BMI, body mass index; MUAC, mid-upper arm circumference; TSFT, triceps skinfold thickness.

Values in bold indicate statistical significance (*p* < 0.05).

^1^ Friedman test, pairwise comparisons with Bonferroni correction (significance: *p* < 0.016).

^2^ Wilcoxon test.

^3^ Paired samples test (for baseline vs. 6th month).

*p = 0.012. **p = 0.005. ***p = 0.003 compared to baseline.

### WFA and WFH *z* scores according to CP subgroups and baseline gastrointestinal symptoms

When analyzed with respect to clinical classification, WFA *z* scores in children with mixed CP [−1.14 (−6.85/1.16) vs. −1.04 (−5.77/0.94), *p* = 0.037] and WFH *z* scores in those with spastic CP [−2.14 (−7.03/2.9) vs. −1.64 (−7/3.37), *p* = 0.002] were significantly improved at 6th month compared to baseline (Table 4).

**Table 4:** WFA and WFH *z* scores according to CP classification and baseline gastrointestinal symptoms in children with CP who received a specialized peptide-based enteral formula.

		WFA *z* score				WFH *z* score			
		*n*	Baseline	6th month	*p*	*n*	Baseline	6th month	*p*
			Median (min/max)	Median (min/max)			Median (min/max)	Median (min/max)	
CP classification									
Clinical classification	Spastic	57	−2 (−6.5/2.32)	−2.17 (−6.48/2.6)	0.417	67	−2.14 (−7.03/2.9)	−1.64 (−7/3.37)	**0**.**002**
	Extrapyramidal	4	−2.1 (−3.61/−0.3)	−1.95 (−3.89/0.35)	0.599	4	0.04 (−1.43/0.64)	−0.18 (−4/0.88)	0.230
	Mixed	12	−1.14 (−6.85/1.16)	−1.04 (−5.77/0.94)	**0**.**037**	14	−1.81 (−7.4/3.23)	−1.23 (−5.61/3.56)	0.173
Functional classification	No activity limitation	10	−2.76 (−6.85/−0.8)	−2.16 (−5.77/−0.64)	**0**.**009**	11	−2.1 (−7.4/1.68)	−1.15 (−5.61/1.69)	0.339
	Mild activity limitation	28	−1.8 (−5.61/1.77)	−2.09 (−6.13/2.19)	0.263	29	−1.67 (−5.48/0.97)	−1.55 (−5.74/2.13)	0.512
	Severe activity limitation	22	−1.72 (−6.22/1.86)	−0.9 (−6.48/1.88)	0.226	28	−2.25 (−7.03/3.23)	−1.6 (−7/3.56)	**0**.**020**
	No functional activity	14	−2.43 (−6.5/2.32)	−2.98 (−6.34/2.6)	0.723	18	−1.2 (−5.67/2.9)	−0.86 (−5.49/3.37)	0.272
Initial gastrointestinal symptoms									
Reflux/regurgitation	No	46	−2.39 (−6.85/1.86)	−2.1 (−5.77/2.22)	0.827	54	−2.12 (−7.4/3.23)	−1.69 (−6.98/3.56)	0.576
	Yes	31	−1.86 (−6.5/2.32)	−2.17 (−6.48/2.6)		35	−1.67 (−6.98/2.9)	−1.22 (−7/3.19)	
Stomachache	No	29	−1.82 (−6.85/1.16)	−2.03 (−5.77/2.22)	0.682	35	−2.02 (−7.4/3.23)	−1.66 (−5.61/3.56)	0.488
	Yes	23	−2.77 (−6.5/2.32)	−2.17 (−6.34/2.6)		26	−1.68 (−7.03/2.9)	−1.38 (−6.98/3.19)	
Discomfort	No	27	−2.27 (−6.85/1.86)	−2.28 (−6.48/2.22)	0.740	31	−1.98 (−7.4/3.23)	−1.66 (−7/3.56)	0.465
	Yes	51	−2 (−6.5/2.32)	−2.17 (−6.34/2.6)		59	−2.1 (−7.03/2.9)	−1.36 (−6.98/3.19)	
Nausea	No	34	−2.39 (−6.85/1.86)	−2.12 (−5.77/2.22)	0.872	39	−2.1 (−7.4/3.23)	−1.9 (−6.98/3.56)	0.484
	Yes	39	−1.88 (−6.5/2.32)	−2.17 (−6.48/2.6)		44	−1.96 (−6.98/2.9)	−1.23 (−7/3.19)	
Retching	No	34	−2.71 (−6.85/1.86)	−2.1 (−6.48/2.22)	0.939	38	−2.1 (−7.4/3.23)	−2.01 (−7/3.56)	0.619
	Yes	44	−1.84 (−6.5/2.32)	−2.17 (−6.34/2.6)		52	−1.97 (−7.03/2.9)	−1.23 (−6.98/3.19)	
Vomiting	No	53	−2.27 (−6.85/1.86)	−2.06 (−6.48/2.22)	0.858	60	−2.12 (−7.4/3.23)	−1.62 (−7/3.56)	0.388
	Yes	25	−1.88 (−5.61/2.32)	−2.17 (−6.13/2.6)		30	−1.89 (−5.54/2.9)	−1.26 (−5.38/3.19)	
Abdominal distension	No	50	−2.39 (−6.5/1.86)	−2.54 (−6.48/2.22)	0.127	57	−2.1 (−7.03/1.91)	−1.9 (−7/3.37)	0.086
	Yes	27	−1.79 (−5.06/2.32)	−1.7 (−4.95/2.6)		31	−1.87 (−5.06/3.23)	−1.15 (−5.38/3.56)	
Diarrhea	No	67	−1.88 (−6.85/2.32)	−2.17 (−6.48/2.6)	0.815	77	−2.1 (−7.4/3.23)	−1.64 (−7/3.56)	0.772
	Yes	11	−2.58 (−5.36/0.35)	−2.33 (−4.04/0.73)		13	−1.32 (−5.38/0.7)	−0.81 (−4/1.8)	
Constipation	No	32	−1.8 (−6.85/1.86)	−1.71 (−5.77/2.19)	0.110	34	−1.36 (−7.4/3.23)	−0.65 (−5.61/3.56)	0.053
	Yes	46	−2.58 (−6.5/2.32)	−2.54 (−6.48/2.6)		56	−2.12 (−7.03/2.9)	−1.78 (−7/3.37)	
Gas	No	41	−2 (−6.85/1.86)	−2.33 (−6.48/2.22)	0.789	44	−2.08 (−7.4/3.23)	−1.4 (−7/3.56)	0.951
	Yes	37	−2.12 (−5.92/2.32)	−2.06 (−5.54/2.6)		45	−1.96 (−7.03/2.9)	−1.57 (−6.98/3.19)	

Repeated measures.

Values in bold indicate statistical significance (*p* < 0.05).

When analyzed with respect to functional classification, WFA *z* scores in children with no activity limitation [−2.76 (−6.85/−0.8) vs. −2.16 (−5.77/−0.64), *p* = 0.009] and WFH *z* scores in those with severe activity limitation [−2.25 (−7.03/3.23) vs. −1.6 (−7/3.56), *p* = 0.020] were significantly improved at 6th month compared to baseline (Table 4).

No significant change was noted in WFA and WFH *z* scores with respect to type of initial individual gastrointestinal symptoms (Table 4).

### Gastrointestinal intolerance symptoms during study visits

**Figure 2:**
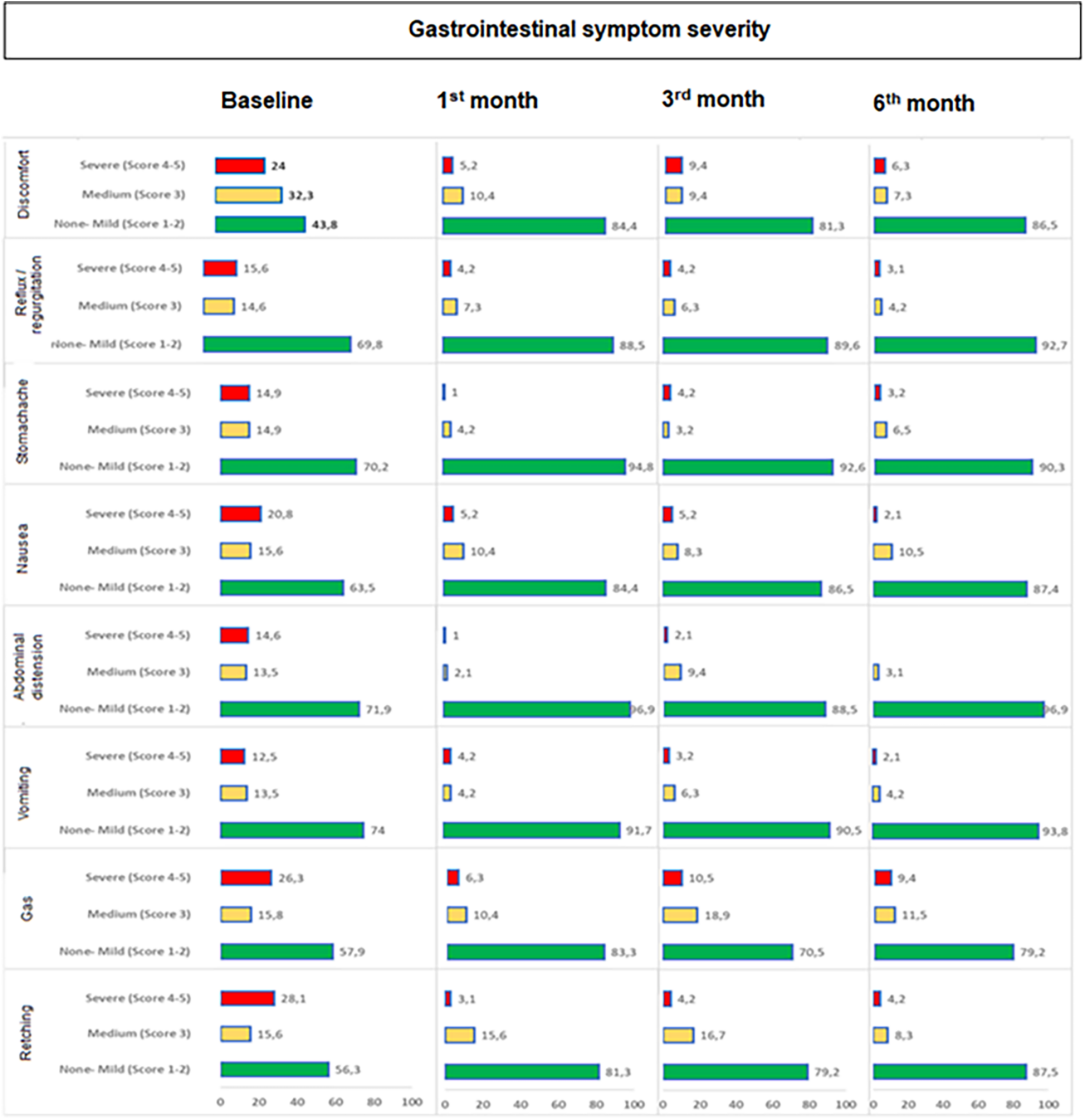
Change in gastrointestinal symptom severity during study visits. Overall, the rate of “severe (score 4−5) symptoms” were significantly decreased, while the rate of “no or mild symptoms” were significantly increased from baseline to 1st month (*p* < 0.001 for each), 3rd month (*p* values ranged 0.002 to <0.001) and 6th month (*p* < 0.001 for each).

Overall, the rate of “severe (score 4–5) symptoms” were significantly decreased, while the rate of “no or mild symptoms” were significantly increased from baseline to 1st month (*p* < 0.001 for each), 3rd month (*p* values ranged 0.002 to <0.001) and 6th month (*p* < 0.001 for each). The improvement (increase in those with no or mild degree symptoms) from baseline was particularly remarkable for discomfort (43.8–86.5%), retching (56.3–87.5%) and abdominal distension (71.9–96.9%) symptoms (Table 5, Figure 2).

**Table 5:** Gastrointestinal intolerance symptom severity and frequency in children with CP who received a specialized peptide-based enteral formula.

Gastrointestinal symptom severity, *n* (%)		Baseline A	1st month B	3rd month C	6th month D	*p* value					
						Total	B vs. A	C vs. A	D vs. A	B vs. C	D vs. C
Discomfort	Score 1–2 (none-mild)	42 (43.8)	81 (84.4)	78 (81.3)	83 (86.5)	**<0**.**001**					
	Score 3 (moderate)	31 (32.3)	10 (10.4)	9 (9.4)	7 (7.3)		**<0**.**001**	**<0**.**001**	**<0**.**001**	0.387	0.414
	Score 4–5 (severe)	23 (24.0)	5 (5.2)	9 (9.4)	6 (6.3)						
Reflux/Regurgitation	Score 1–2 (none- mild)	67 (69.8)	85 (88.5)	86 (89.6)	89 (92.7)	**<0**.**001**					
	Score 3 (moderate)	14 (14.6)	7 (7.3)	6 (6.3)	4 (4.2)		**<0**.**001**	**<0**.**001**	**<0**.**001**	0.501	0.802
	Score 4–5 (severe)	15 (15.6)	4 (4.2)	4 (4.2)	3 (3.1)						
Stomachache	Score 1–2 (none- mild)	66 (70.2)	91 (94.8)	88 (92.6)	84 (90.3)	**<0**.**001**					
	Score 3 (moderate)	14 (14.9)	4 (4.2)	3 (3.2)	6 (6.5)		**<0**.**001**	**<0**.**001**	**<0**.**001**	0.968	0.941
	Score 4–5 (severe)	14 (14.9)	1 (1)	4 (4.2)	3 (3.2)						
Nausea	Score 1–2 (none-mild)	61 (63.5)	81 (84.4)	83 (86.5)	83 (87.4)	**<0**.**001**					
	Score 3 (moderate)	15 (15.6)	10 (10.4)	8 (8.3)	10 (10.5)		**<0**.**001**	**<0**.**001**	**<0**.**001**	0.347	0.822
	Score 4–5 (severe)	20 (20.8)	5 (5.2)	5 (5.2)	2 (2.1)						
Abdominal Distension	Score 1–2 (none- mild)	69 (71.9)	93 (96.9)	85 (88.5)	93 (96.9)	**<0**.**001**					
	Score 3 (moderate)	13 (13.5)	2 (2.1)	9 (9.4)	3 (3.1)		**<0**.**001**	**<0**.**001**	**<0**.**001**	0.041	0.134
	Score 4–5 (severe)	14 (14.6)	1 (1)	2 (2.1)	-						
Vomiting	Score 1–2 (none- mild)	71 (74)	88 (91.7)	86 (90.5)	90 (93.8)	**<0**.**001**					
	Score 3 (moderate)	13 (13.5)	4 (4.2)	6 (6.3)	4 (4.2)		**<0**.**001**	**0**.**001**	**<0**.**001**	0.429	0.313
	Score 4–5 (severe)	12 (12.5)	4 (4.2)	3 (3.2)	2 (2.1)						
Gas	Score 1–2 (none- mild)	55 (57.9)	80 (83.3)	67 (70.5)	76 (79.2)	**<0**.**001**					
	Score 3 (moderate)	15 (15.8)	10 (10.4)	18 (18.9)	11 (11.5)		**<0**.**001**	**0**.**002**	**<0**.**001**	0.032	0.233
	Score 4–5 (severe)	25 (26.3)	6 (6.3)	10 (10.5)	9 (9.4)						
Retching	Score 1–2 (none- mild)	54 (56.3)	78 (81.3)	76 (79.2)	84 (87.5)	**<0**.**001**					
	Score 3 (moderate)	15 (15.6)	15 (15.6)	16 (16.7)	8 (8.3)		**<0**.**001**	**<0**.**001**	**<0**.**001**	0.594	0.211
	Score 4–5 (severe)	27 (28.1)	3 (3.1)	4 (4.2)	4 (4.2)						
Gastrointestinal symptom frequency, *n* (%)		Baseline A	1st month B	3rd month C	6th month D	*p* value					
						Total	B vs. A	C vs. A	D vs. A	B vs. C	D vs. C
Discomfort	None-once a month	30 (32.6)	70 (76.1)	70 (76.1)	72 (78.3)	**<0**.**001**					
	On a weekly basis	36 (39.1)	15 (16.3)	13 (14.1)	12 (13)		**<0**.**001**	**<0**.**001**	**<0**.**001**	0.388	0.376
	Ona daily basis	26 (28.3)	7 (7.6)	9 (9.8)	8 (8.7)						
Reflux/Regurgitation	None-once a month	63 (68.5)	74 (80.4)	78 (84.8)	78 (84.8)	**<0**.**001**					
	On a weekly basis	14 (15.2)	11 (12)	12 (13)	10 (10.9)		**0**.**001**	**<0**.**001**	**<0**.**001**	0.235	0.571
	Ona daily basis	15 (16.3)	7 (7.6)	2 (2.2)	4 (4.3)						
Stomachache	None-once a month	59 (65.6)	80 (88.9)	83 (92.3)	80 (88.9)	**<0**.**001**					
	On a weekly basis	23 (25.5)	8 (8.9)	4 (4.4)	7 (7.8)		**<0**.**001**	**<0**.**001**	**<0**.**001**	0.531	0.587
	Ona daily basis	8 (8.9)	2 (2.2)	3 (3.3)	3 (3.3)						
Nausea	None-once a month	50 (54.3)	69 (75)	72 (78.3)	71 (77.2)	**<0**.**001**					
	On a weekly basis	19 (20.7)	16 (17.4)	14 (15.2)	16 (17.4)		**<0**.**001**	**<0**.**001**	**<0**.**001**	0.471	0.758
	Ona daily basis	23 (25)	7 (7.6)	6 (6.5)	5 (5.4)						
Abdominal Distension	None-once a month	64 (70.3)	86 (94.5)	82 (90.1)	83 (91.2)	**<0**.**001**					
	On a weekly basis	19 (20.9)	3 (3.3)	8 (8.8)	7 (7.7)		**<0**.**001**	**<0**.**001**	**<0**.**001**	0.330	0.792
	Ona daily basis	8 (8.8)	2 (2.2)	1 (1.1)	1 (1.1)						
Vomiting	None-once a month	64 (71.1)	79 (87.8)	77 (85.6)	79 (87.8)	**<0**.**001**					
	On a weekly basis	16 (17.8)	7 (7.8)	9 (10)	8 (8.9)		**0**.**001**	**0**.**003**	**0**.**002**	0.720	0.397
	Ona daily basis	10 (11.1)	4 (4.4)	4 (4.4)	3 (3.3)						
Gas	None-once a month	45 (49.4)	63 (69.2)	62 (68.1)	62 (68.1)	**<0**.**001**					
	On a weekly basis	24 (26.4)	17 (18.7)	16 (17.6)	19 (20.9)		**<0**.**001**	**0**.**002**	**<0**.**001**	0.333	0.749
	Ona daily basis	22 (24.2)	11 (12.1)	13 (14.3)	10 (11)						
Retching	None-once a month	43 (46.2)	64 (68.8)	70 (75.3)	72 (77.4)	**<0**.**001**					
	On a weekly basis	27 (29.1)	20 (21.5)	16 (17.2)	13 (14)		**<0**.**001**	**<0**.**001**	**<0**.**001**	0.560	0.508
	Ona daily basis	23 (24.7)	9 (9.7)	7 (7.5)	8 (8.6)						

Gastrointestinal symptom frequency scores of “1 to 6” were re-grouped to include none-once a month (score 1 and 2), on a weekly basis (score 3 and 4) and on a daily basis (score 5 and 6) groups.

Values in bold indicate statistical significance (*p* < 0.05).

Friedman test and Wilcoxon test for pairwise comparisons (significance: *p* < 0.008 after Bonferroni correction).

**Figure 3:**
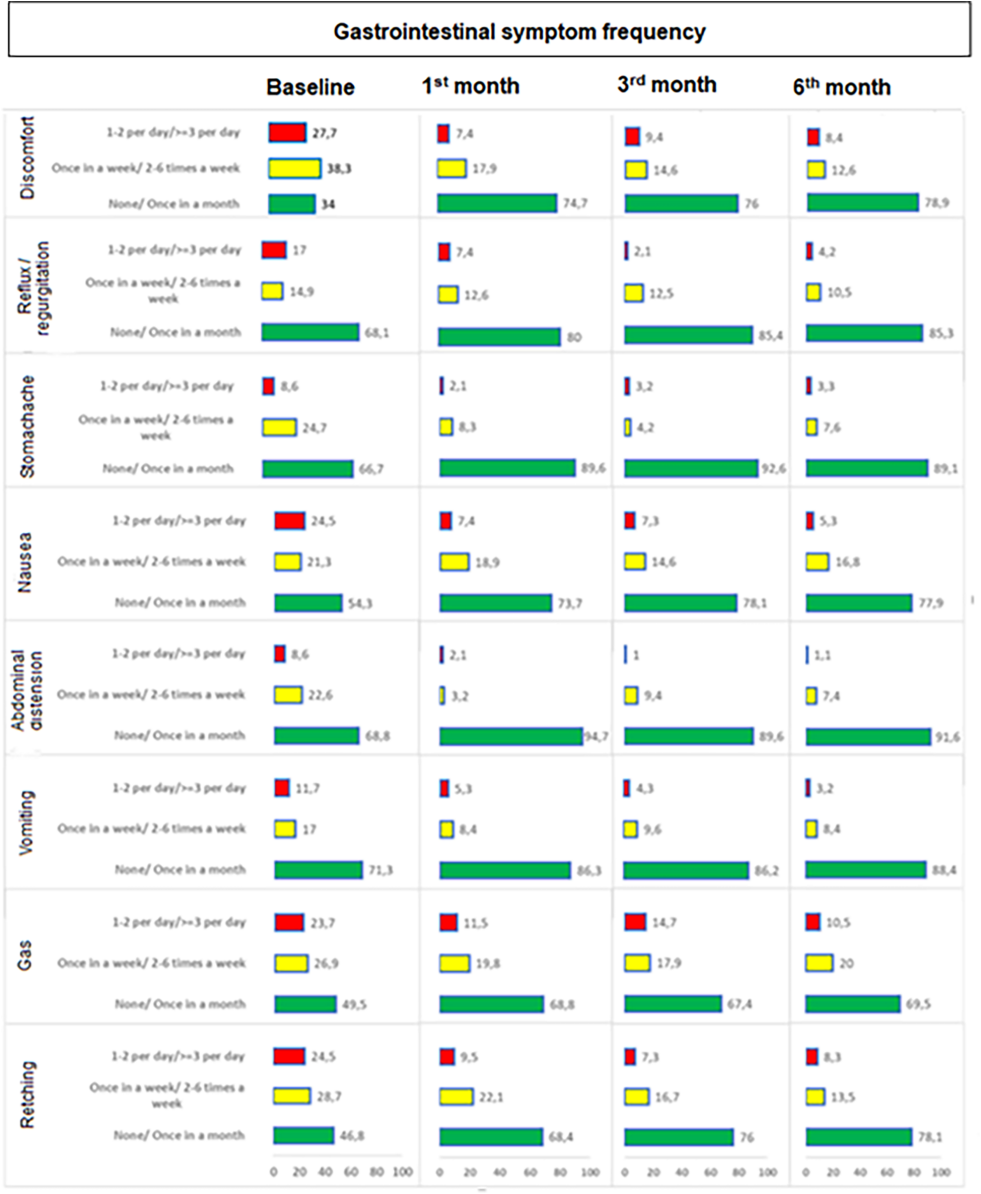
Change in gastrointestinal symptom frequency during study visits. The percentage of children who experience symptoms on a daily basis significantly decreased along with significant increase in the percentage of children with no or once monthly symptoms baseline to 1st month (*p* values ranged 0.001 to <0.001 for each), 3rd month (*p* values ranged 0.003 to <0.001) and 6th month (*p* values ranged 0.002 to <0.001).

The percentage of children who experience symptoms on a daily basis significantly decreased along with significant increase in the percentage of children with no or once monthly symptoms baseline to 1st month (*p* values ranged 0.001 to <0.001 for each), 3rd month (*p* values ranged 0.003 to <0.001) and 6th month (*p* values ranged 0.002 to <0.001). In general, improvement in frequency occurred within one month and was sustained through 6 months with increase in the percentage of children with lesser symptom frequency to ∼70% or 75% depending on symptom which stayed at that level throughout. The improvement (decrease in those who experience symptoms on a daily basis) from baseline was particularly remarkable for discomfort (28.3 to 8.7%), nausea (25.0 to 5.4%), retching (24.7 to 8.6%) and gas (24.2 to 11.0%) symptoms (Table 5, Figure 3).

### Defecation frequency and stool patterns during study visits

When compared to baseline, 1st month, 3rd month and 6th month assessments revealed significant decrease in the likelihood of Type-1/Type-2 (constipation) stool pattern (from 31.6% at baseline to 7.4%, 2.1% and 3.2%, respectively, *p* < 0.001 for each) (Table 6).

**Table 6:** Defecation frequency and stool patterns in children with CP who received a specialized peptide-based enteral formula.

	Baseline A	1st month B	3rd month C	6th month D	*p* value						
					Total	B vs. A	C vs. A	D vs. A	C vs. B	D vs. B	C vs. D
Stool pattern, *n* (%)											
Type-1/Type-2 *(constipation)*	30 (31.6)	7 (7.4)	2 (2.1)	3 (3.2)	**<0**.**001**						
Type-3/Type-4 *(normal)*	27 (28.4)	34 (35.8)	34 (35.8)	35 (36.8)		**<0**.**001**	**<0**.**001**	**<0**.**001**	0.102	<0.001	0.513
Type-5/Type-6/Type-7 *(diarrhea)*	38 (40.0)	54 (56.8)	59 (62.1)	57 (60.0)							
Defecation frequency, *n* (%)											
Once a week or less	35 (36.5)	8 (8.3)	12 (12.5)	8 (8.3)	**0**.**001**						
Once a day/once in every 2–3 days	41 (42.7)	68 (70.8)	71 (74)	74 (77.1)		**<0**.**001**	0.049	0.012	0.048	0.273	0.225
At least 2–3 times per day	20 (20.8)	20 (20.8)	13 (13.5)	14 (14.6)							
Normal defecation frequency^a^ in children with symptoms, *n* (%)											
Reflux/regurgitation	17 (44.7)	27 (71.1)	28 (73.7)	29 (76.3)	**0**.**008**	**0**.**001**	0.072	0.019	0.366	1.000	0.180
Stomachache	11 (39.3)	21 (75.0)	21 (75)	19 (67.9)	**<0**.**001**	**<0**.**001**	**0**.**007**	**0**.**002**	0.034	1.000	0.014
Discomfort	29 (46)	47 (74.6)	47 (74.6)	49 (77.8)	**<0**.**001**	**<0**.**001**	0.078	0.010	0.011	0.180	0.058
Nausea	22 (46.8)	32 (68.1)	34 (72.3)	37 (78.7)	**0**.**004**	**0**.**001**	0.041	0.027	0.317	0.439	0.655
Retching	26 (46.4)	40 (71.4)	44 (78.6)	48 (85.7)	**0**.**008**	**0**.**003**	0.051	0.023	0.346	0.617	0.480
Vomiting	16 (50)	23 (71.9)	24 (75)	25 (78.1)	**0**.**049**	0.013	0.201	0.050	0.317	1.000	0.083
Abdominal distension	14 (40)	25 (71.4)	25 (71.4)	28 (80)	**<0**.**001**	**<0**.**001**	**0**.**001**	**<0**.**001**	0.285	0.763	0.257
Residue (for gastrostomy patients)	7 (70)	8 (80)	9 (90)	9 (90)	0.190	0.083	1.000	1.000	0.083	0.083	1.000
Gas	20 (42.6)	35 (74.5)	37 (78.7)	36 (76.6)	**<0**.**001**	**<0**.**001**	**0**.**003**	**0**.**001**	0.059	0.197	0.180

Values in bold indicate statistical significance (*p* < 0.05).

Friedman test and Wilcoxon test for pairwise comparisons (significance: *p* < 0.008 after Bonferroni correction).

^a^ Once a day/once in every 2–3 days.

The rate of normal (once a day or in every 2–3 days) defecation frequency was also increased from 42.7% at baseline to 70.8% at 1st month (*p* < 0.001) and this increase was maintained at 3rd and 6th month visits, particularly in the presence of stomachache (from 39.3 to 75% at 3rd month, *p* = 0.007 and to 67.9% at 6th month, *p* = 0.002), abdominal distension (from 40.0 to 71.4% and 80.0%, *p* = 0.001 and *p* < 0.001, respectively) and gas (from 42.6 to 78.7% and 76.6%, *p* = 0.003 and *p* = 0.001, respectively) (Table 6).

### Parent assessment on feeding and stool patterns

Most of parents reported that peptide-based enteral formula was associated with normalization of bowel movements (>85%) via decreasing the likelihood of increased bowel movements (>68%) as well as the constipation (>76%), with no vomiting after feeding (>82%) and an improved general status of the child (>79%), regardless of the follow up visit (Table 7).

**Table 7:** Parent assessment on feeding and stool patterns and parental satisfaction with the nutritional product in children with CP.

Parental satisfaction—Formula Satisfaction Questionnaire, *n* (%)		1st month	3rd month	6th month
How satisfied were you with the nutritional product overall?	Satisfied	72 (79.1)	76 (80)	84 (90.3)
	Moderately satisfied	17 (18.7)	18 (18.9)	9 (9.7)
	Not satisfied	2 (2.2)	1 (1.1)	—
Would you like to continue using the nutritional product?	Yes	83 (91.2)	86 (90.5)	88 (94.6)
	Maybe	8 (8.8)	8 (8.4)	3 (3.2)
	No	—	1 (1.1)	2 (2.2)
What is your favorite feature of the nutritional product?	Easily available in pharmacies	53 (55.2)	58 (60.4)	55 (57.3)
	Product box/packaging	33 (34.4)	49 (51.0)	40 (41.7)
	Ease of use of the product	59 (61.5)	54 (56.3)	69 (71.9)
	The consistency of the product	36 (37.5)	52 (54.2)	46 (47.9)
	The smell of the product	33 (34.4)	37 (38.5)	42 (43.8)
	Nursing service provided	14 (14.6)	24 (25)	28 (29.2)
	Other	13 (13.5)	13 (13.5)	9 (9.4)
Parent assessment on feeding and stool patterns—Feeding and Stool Patterns Questionnaire, *n* (%)		1st month	3rd month	6th month
How was your child's general status while using the nutritional product?	Good	72 (79.1)	81 (85.3)	81 (87.1)
	Moderate	18 (19.8)	14 (14.7)	12 (12.9)
	Poor	1 (1.1)	—	—
Did your child have any problems while taking the nutritional product?	No	89 (97.8)	89 (93.7)	88 (94.6)
	Yes	2 (2.2)	6 (6.3)	5 (5.4)
My child vomited after feeding	Always Often	3 (3.3)	4 (4.3)	4 (4.3)
	Sometimes	13 (14.4)	11 (12)	10 (10.8)
	Rarely Never	74 (82.2)	77 (83.7)	79 (84.9)
My child's bowel movements are in order	Always Often	65 (71.4)	67 (70.5)	76 (81.7)
	Sometimes	16 (17.6)	14 (14.7)	10 (10.8)
	Rarely Never	10 (11)	14 (14.7)	7 (7.5)
There were days when bowel movements were too much	Always Often	6 (6.7)	6 (6.4)	8 (8.6)
	Sometimes	15 (16.7)	20 (21.3)	21 (22.6)
	Rarely Never	69 (76.7)	68 (72.3)	64 (68.8)
My child is constipated	Always Often	10 (11.1)	10 (10.8)	5 (5.4)
	Sometimes	11 (12.2)	8 (8.6)	8 (8.6)
	Rarely Never	69 (76.7)	75 (80.6)	80 (86)

### Parent's satisfaction with the nutritional product

Majority of parents were satisfied with the study formula (97.8% at 1st month visit, 98.9% at 3rd month visit and 100.0% at 6th month visit), reported no problems (97.8%, 93.7% and 94.6%, respectively) and they wished to continue using the enteral formula (91.2%, 90.5% and 94.6%, respectively) (Table 7).

The favorite feature of the product was considered to be ease of use (by 61.5%, 56.3% and 71.9% of parents at 1st, 3rd and 6th month visits, respectively) and its availability in pharmacies (by 55.2%, 60.4% and 57.3% of parents at 1st, 3rd and 6th month visits, respectively) (Table 7).

### Laboratory findings and adverse events

Other than significantly increased percentage of patients with high serum albumin (13.6 vs. 47.5%, *p* < 0.001), no significant change was noted in blood analysis findings during follow up visits. In total, 28 adverse events were reported in 22 children during study period, and the most frequently reported AEs were death (*n* = 6), pneumonia (*n* = 6), discomfort with PEG (*n* = 6) and upper respiratory infection (*n* = 5) (Table 8).

**Table 8:** Laboratory findings and adverse events during nutritional support in children with CP.

		Baseline	3rd month	6th month	*p* value^1^
Blood analysis					
Leukocyte (*n* = 60), median (min/max)		8.6 (3/29.7)	8.2 (2.5/74.6)	8.3 (3.3/25.6)	
*n* (%)	Low	5 (8.3)	3 (5)	2 (3.3)	0.254
	Normal	42 (70)	50 (83.3)	45 (75)	
	High	13 (21.7)	7 (11.7)	13 (21.7)	
Hemoglobin (*n* = 68), median (min/max)		12.9 (7.1/16.9)	13.0 (9.3/16.5)	13.3 (6.5/15.9)	
*n* (%)	Low	8 (11.8)	9 (13.2)	4 (5.9)	0.058
	Normal	52 (76.4)	49 (72.1)	52 (76.5)	
	High	8 (11.8)	10 (14.7)	12 (17.6)	
Hematocrit (*n* = 68), median (min/max)		38 (23/50)	38 (4.6/48)	39 (23/45.9)	
*n* (%)	Low	7 (10.3)	5 (7.4)	3 (4.4)	0.104
	Normal	57 (83.8)	54 (79.4)	59 (86.8)	
	High	4 (5.9)	9 (13.2)	6 (8.8)	
Serum albumin (*n* = 59), median (min/max)		4 (3.0/6.0)	4.2 (3/7.3)	4.3 (3.3/7.5)	
*n* (%)	Low	3 (5.1)	1 (1.7)	1 (1.7)	**<0**.**001**
	Normal	48 (81.3)	48 (81.4)	30 (50.8)	
	High	8 (13.6)	10 (16.9)	28 (47.5)	
Total protein (*n* = 50), median (min/max)		6.7 (1.6/8.1)	6.8 (3.0/8.1)	7.0 (3.0/8.3)	
*n* (%)	Low	11 (22)	6 (12)	5 (10)	0.156
	Normal	38 (76)	44 (88)	43 (86)	
	High	1 (2)	—	2 (4)	
Adverse events	*n* (%)				
Patients with AEs	22 (22.9)				
Death	6 (6.3)				
Pneumonia	6 (6.3)				
Discomfort with the PEG^a^	6 (6.3)				
Upper respiratory infection	5 (5.2)				
Gastrointestinal bleeding	1 (1.0)				
Increase in seizures	1 (1.0)				
Sepsis	1 (1.0)				
Occluded tracheostomy	1 (1.0)				
Viscous saliva	1 (1.0)				
Total number of AEs	28^2^				

Values in bold indicate statistical significance (*p* < 0.05).

^a^ Obstruction, redness, food leakage, increased arrivals, leakage, bleeding.

^1^ Friedman test and pairwise comparisons with the Wilcoxon test (*p*-value after Bonferroni correction: 0.017).

^2^ More than one event occurred in 5 patients.

## Discussion

Our findings revealed that use of a specialized peptide-based formula containing MCT (Pediasure® peptide in majority of cases) in children with CP and feeding intolerance on previous tube feeding with standard enteral formula was associated with improved anthropometrics, amelioration in gastrointestinal intolerance symptoms both in terms of severity and frequency, normalization of defecation frequency and stool patterns and a high parental satisfaction with the formula. In general, improvement in frequency occurred within one month and was sustained through 6 months with increase in the percentage of children with lesser symptom frequency to ∼70% or 75% depending on symptom which stayed at that level throughout.

Spastic CP as accompanied with severe or total functional limitation was the leading diagnosis in our study population, while gastrointestinal discomfort, constipation and retching were the most common gastrointestinal intolerance symptoms recorded at baseline. Similarly, in a study among 1,108 children with CP from Turkey, the quadriplegic spastic CP was reported in majority of children in addition to Gross Motor Function Classification System (GMFCS) level V motor dysfunction, co-morbid gastrointestinal problems (constipation, lack of appetite and difficulty in swallowing) and malnutrition, especially in those with higher levels of gross motor dysfunction (7). Data from the multi-country PURPLE N (Profiling Children and Youth with Cerebral Palsy in Relation to Feeding and Nutrition) study, including Turkey, also revealed the severe form of CP (spastic CP in 84%, quadriplegia in 55%, and GMFCS level VI-V in 50%) along with gastrointestinal problems (i.e., constipation, gastrointestinal reflux, vomiting and retching) in most of children and a higher risk of malnutrition particularly in those with higher GMFCS level (31).

In fact, prevalence of malnutrition reported by different studies in children with CP ranges from 40% to 90%, depending on the study population and anthropometric tool (32–35). Previous multicenter studies in our country also revealed a considerable difference in the prevalence of malnutrition in children with CP, which was reported to be 57.2%, 92.6% to 94.3% and 91.3%, based on physicians' clinical judgment, Gomez classification of WFA percentiles and Waterlow classification of HFA percentiles, respectively (7, 36). Hence, use of growth charts for general pediatric population for anthropometric assessment in children with CP is considered to be associated with a risk of overestimating malnutrition in these children (7, 32, 37). Indeed, while anthropometric references exist for children with CP, they are not currently recommended because they only allow to know the growth from children with different degrees of severity, not the desirable growth, so the nutritional objective is restricted (38, 39).

Nonetheless, supporting the consideration of non-ambulatory status amongst the strongest risk factors for malnutrition in children with CP, several studies indicated association of higher levels of gross motor impairment with an increased risk of malnutrition and anthropometric deficits in curves in children with CP (7, 12, 18, 31, 40, 41). In addition, children with CP are suggested to have significantly lower anthropometric values in case of severe comorbidities and gastrointestinal problems, emphasizing the potential role of nutritional assessment and management as part of their overall care (42). The favorable efficacy and tolerability profile of peptide-based enteral formula in our children with severely disabled CP and intolerance on previous tube feeding with standard enteral formula is important in this regard.

Stunted growth and underdeveloped fat-free mass are considered to be more pronounced in more severe forms of CP, and therefore using screening tools for growth and fat mass is suggested to be more efficient in children with higher the GMFCS levels (37, 43). Notably, the combined use of MUAC, age, and GMFCS level is recommended in accurate prediction of weight in children with CP as well as the use of circumferences, primarily MUAC, in combination with other methods (i.e., TSFT) in the anthropometric assessment (37, 44, 45). Accordingly, our findings related to achievement of significantly improved MUAC *z* scores and TSFT *z* scores, as the highly sensitive measures of diagnosing malnutrition in the setting of CP, seem consistent with the multimodal effects of peptide-based enteral formula in terms of bone, muscle, and the adipose panicle (the energetic balance) (45–48).

In addition, WFH *z* scores were significantly improved at 6th month in our overall study population as well as in the subgroups of children with “spastic CP and severe activity limitation”, while WFA *z* scores were significantly improved at 6th month in those with “mixed CP and no activity limitation”. These findings seem notable given the increased likelihood of feeding difficulties in children with CP who have more severe motor impairment (i.e., tetraparesis) and more varied motor symptoms (i.e., mixed CP) (4). Other studies also reported the change in nutritional improvement according to the muscle tonicity in children with CP on PEG tube feeding, such as improved BMI for-age z-score and percent ideal body weight in the hypertonic group but not in the hypotonic group (49, 50). This emphasizes the possibility of different growth patterns and thus different nutritional support needs depending on the CP type (49, 50). Also, in a study addressing the factors related to caregiver burden on activities of daily living (ADLs) in 69 children with CP, GMFCS grade and intellectual disability were reported to be associated increased caregiver burden score, whereas, greater difficulty in performing ADLs was noted with lower weight *z* scores, BMI *z* scores and fat mass, regardless of the degree of GMFCS and intellectual disability (51). Accordingly, in addition to the degree of clinical impairment, nutritional status is also considered a key factor affecting the caregiver's difficulty in performing the ADLs in children with CP (51).

Immobile children with 11 kcal/cm height/day energy need comprised the majority of our study population in each follow up visit (72.3%, 77.7% and 82.1%, respectively) and the median values for TEE in these children were 1,168 kcal/day at baseline and 1,199 kcal/day at 1st month. Likewise, in a study among 13 children with CP, nutritional support for four weeks was reported to reveal a significant increase in TEE (from 1,121 kcal/day at baseline to 1,189 kcal/day at 30 days) (52). In this regard, our findings support the likelihood of normalization of TEE via an adequate energy intake in children with CP, which is lower than in healthy children due to adaptation of being fed with low energy diets over a prolonged period of time (52, 53).

Although, use of PEG (standard polymeric enteral formula) in children with CP has been reported to ameliorate malnutrition with improved anthropometrics, achievement of most of the catch-up growth and nutritional correction in approximately 6 months after PEG tube insertion (24, 49, 54, 55), a substantial population of children with CP and enteral tube feeding are affected by persistent feeding intolerance (56, 57). In this regard, our findings highlight the evaluation of gastrointestinal problems together with nutritional status and neurological and neuromuscular impairment in children with CP, since the concomitant presence of constipation, diarrhea, gastroesophageal reflux and feeding intolerance may necessitate alteration in formula and feeding regimens (18, 58). Moreover, selecting the appropriate type of enteral formula is of critical importance in maintaining or recovering the nutritional status in children with CP, as a promising strategy to overcome feeding intolerance and to reduce the related adverse health outcomes (i.e., increased risk of severe illness, mortality and nosocomial infections) (15, 49, 59).

In a systematic review of 15 randomized clinical trials on nutritional and dietary interventions in children with CP, the authors suggested that certain dietary and nutritional interventions offer potential benefits in clinical improvement, such as use of whey-based or pectin-enriched enteral formulas for gastroesophageal reflux, supplementation with lipid mixture or diet with high-density energy for improvements in anthropometric measures, supplementation with probiotics, prebiotics, symbiotics or magnesium for constipation and use of a nutritional support system for gross motor function (60). However, while authors indicated that some promising dietary and nutritional interventions may promote important clinical improvements for patients with CP, they also emphasized that the evidence is weak, due to availability of few published clinical trials along with many methodological errors, leading to a high risk of bias (60).

In our children with severely disabled CP and gastrointestinal intolerance, peptide-based enteral formula ameliorated gastrointestinal symptom severity and frequency starting from the 1st month of nutritional support. The improvement in symptom severity was particularly remarkable for discomfort, retching and abdominal distension, while the improvement in symptom frequency was more pronounced for discomfort, nausea, retching and gas. Hence, our findings support the likelihood of protein composition in enteral formulas to ameliorate the gastrointestinal symptoms among gastrostomy-fed children with CP, possibly in relation to effects on gastric emptying and dysmotility (17, 61). It has also been suggested that children with CP, frequently having concomitant gastrointestinal problems including foregut dysmotility, may be more sensitive to type of protein in the meal than healthy children (17, 62).

The unique characteristics of MCTs such as not requiring a complex process of digestion and their facile absorption without the need for bile or pancreatic enzymes confer significant advantage over most other lipid molecules, particularly in the setting of gastrointestinal disorders (63, 64). Besides the amelioration of gastrointestinal intolerance symptoms, peptide-based enteral formula containing MCTs also revealed additional benefits in terms of normalization of bowel movements with a significant decrease in type 1 and a significant increase in type 4 stool patterns in our children. These effects appeared starting from the 1st month of clinical nutrition and maintained throughout the follow up, even in the presence of stomachache, abdominal distension and gas. This seems notable given that chronic constipation in CP has a significant impact on child's well-being, as can be associated with gastrointestinal manifestations (recurrent vomiting, abdominal discomfort and early satiety, compromising dietary intake), urinary symptoms (poorly voiding bladder, recurrent urinary tract infection and deterioration of vesicoureteric reflux) and impaired quality of life (11, 58, 59, 65).

Majority of parents in the current study were satisfied with the study formula, reported significantly improved bowel movements and stool patterns and general status of the child with no adverse reactions and they wished to continue using it. Similarly, evidence of caregiver satisfaction with gastrostomy tube feeding was reported in the majority of studies, including PEG-fed children with CP, regarding the ease of feeding, improvement in child's disposition and nutrition, enhanced child's comfort and abilities, less stress and low risk of long-term adverse events (6, 11, 25, 65, 66).

In addition, a high degree of agreement was noted in child feeding and stool patterns as determined by parents and clinicians in the current study, which is important given that family-assessed measurements or family reports, as recently become more popular and important, are considered to be advantageous in terms of providing data not limited to a clinical setting or a visit-time (67, 68).

Given that the nutritional status of children with CP is affected by several factors inherent to their own condition and those beyond dietary intake, such as GMFCS level, oral-motor dysfunction, feeding skills, gastrointestinal disorders, physical activity levels and altered energy requirements, multidisciplinary monitoring and evaluation of nutrition support for children with CP has an important role in timely identification and management of nutritional status and potential complications (28, 58, 59).

Major strength of the present study seems to be availability of a comprehensive assessment of nutritional and gastrointestinal data in a large-scale cohort of children with CP as well as use of a specialized peptide-based enteral tube feeding in the presence of gastrointestinal intolerance symptoms. Certain limitations to this study should be considered. First, due to observational nature, non-randomized allocation and thereby the likelihood of main selection bias and confounding is possible. Second lack of data on co-morbid gastrointestinal dysfunction such as dysphagia, gastroesophageal reflux and oral motor dysfunction is another limitation which otherwise would extend the knowledge achieved in the current study.

## Conclusion

In conclusion, our findings revealed that choice of a specialized peptide-based formula containing MCT in provision of enteral tube feeding among children with CP and feeding intolerance on previous standard enteral formula is an effective strategy that leads to improved anthropometrics, amelioration of gastrointestinal intolerance, and normalization of bowel movements along with a high parental satisfaction. Our findings suggest that nutritional and gastrointestinal problems should concomitantly be assessed in children with CP and those with concomitant gastrointestinal problems may benefit from provision of enteral tube feeding with use of a specialized peptide-based enteral formula. Hence, appropriate identification of nutritional support needs depending on the clinical and functional CP types is important in children with CP as well as the timely recognition of feeding intolerance that may necessitate alteration in formula and feeding regimens for maintaining or recovering the nutritional status. The utility of specialized peptide-based enteral formulas in enteral tube feeding among children with CP should be further addressed by longer term and larger scale studies in terms of nutritional, gastrointestinal and survival outcomes.

## Data Availability

The original contributions presented in the study are included in the article/Supplementary Material, further inquiries can be directed to the corresponding author.
