# Supplementary figures and images for: Use of a specialized peptide-based enteral formula containing medium-chain triglycerides for enteral tube feeding in children with cerebral palsy and previous tube feeding intolerance on standard enteral formula: a prospective observational TolerUP study
Source: Front Pediatr. 2025 Feb 12;13:1448507. doi: 10.3389/fped.2025.1448507 (PMC11861557; doi:10.3389/fped.2025.1448507)

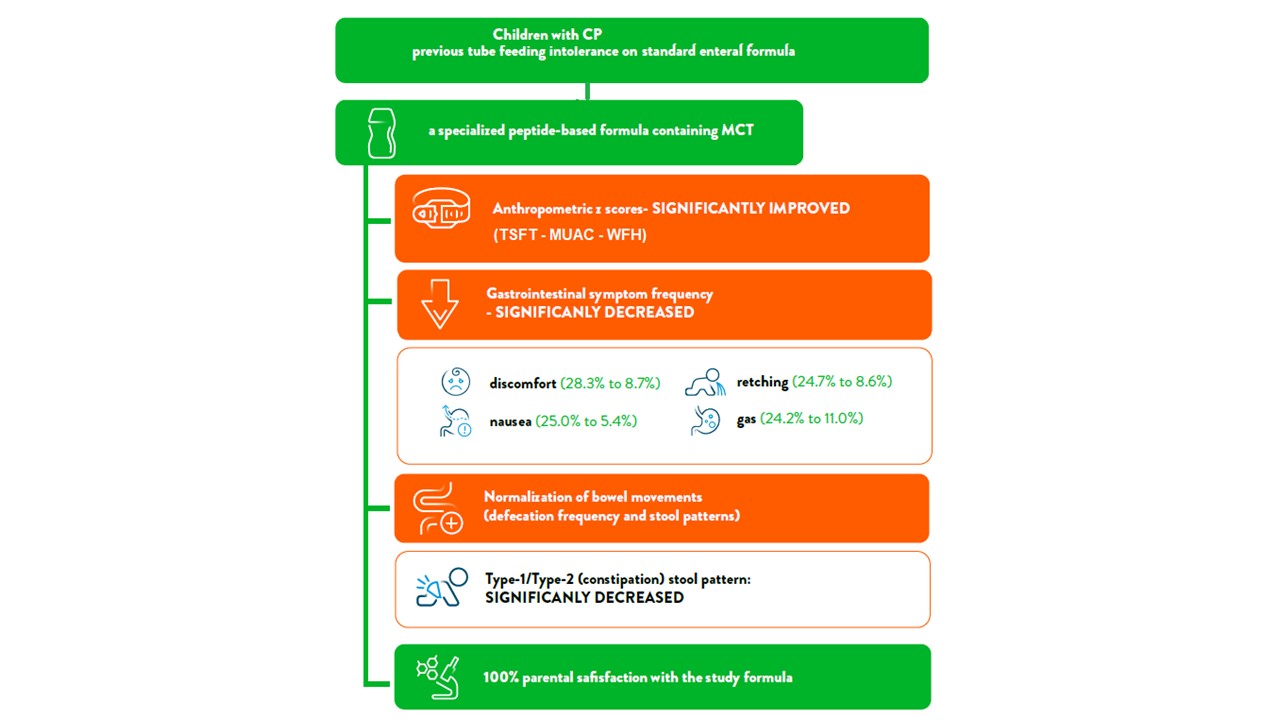

Supplement: Supplementary file 1 [file Image1.jpeg]
